# Novel *DNMT3A* Germline Variant in a Patient with Multiple Paragangliomas and Papillary Thyroid Carcinoma

**DOI:** 10.3390/cancers12113304

**Published:** 2020-11-09

**Authors:** Sara Mellid, Javier Coloma, Bruna Calsina, María Monteagudo, Juan M. Roldán-Romero, María Santos, Luis J. Leandro-García, Javier Lanillos, Ángel M. Martínez-Montes, Cristina Rodríguez-Antona, Cristina Montero-Conde, Joaquín Martínez-López, Rosa Ayala, Xavier Matias-Guiu, Mercedes Robledo, Alberto Cascón

**Affiliations:** 1 Hereditary Endocrine Cancer Group, Spanish National Cancer Research Centre (CNIO), 28029 Madrid, Spain; smellid@cnio.es (S.M.); bcalsina@cnio.es (B.C.); mmonteagudo@cnio.es (M.M.); jmroldan@cnio.es (J.M.R.-R.); msantosr@cnio.es (M.S.); ljleandro@cnio.es (L.J.L.-G.); jlanillos@cnio.es (J.L.); ammontes@cnio.es (Á.M.M.-M.); crodriguez@cnio.es (C.R.-A.); cmontero@cnio.es (C.M.-C.); mrobledo@cnio.es (M.R.); 2 Structural Biology Programme, Spanish National Cancer Research Centre (CNIO), 28029 Madrid, Spain; jcoloma@cnio.es; 3 Centro de Investigación Biomédica en Red de Enfermedades Raras (CIBERER), 28029 Madrid, Spain; 4 Department of Hematology, Hospital 12 de Octubre (‘i+12’), 28041 Madrid, Spain; jmarti01@med.ucm.es (J.M.-L.); rosam.ayala@salud.madrid.org (R.A.); 5 Haematological Malignancies Clinical Research Unit, Spanish National Cancer Research Centre (CNIO), 28029 Madrid, Spain; 6 Complutense University of Madrid, 28040 Madrid, Spain; 7 Centro de Investigación Biomédica en Red de Cáncer (CIBERONC), 28029 Madrid, Spain; fjmatiasguiu.lleida.ics@gencat.cat; 8 Department of Pathology and Molecular Genetics, Hospital Universitari Arnau de Vilanova, University of Lleida, IRBLleida, 25198 Lleida, Spain; 9 Department of Pathology, Hospital Universitari de Bellvitge, IDIBELL, l’Hospitalet de Llobregat, 08908 Barcelona, Spain

**Keywords:** paraganglioma, *DNMT3A*, germline variant, papillary thyroid carcinoma

## Abstract

**Simple Summary:**

The use of next generation technologies has helped to unravel the genetics of rare inherited diseases, facilitating the discovery of new susceptibility genes. Nonetheless, the sequencing of all protein-coding genes of an individual may lead to doubtful assignments of causality for non-pathological variants, so it is mandatory to perform comprehensive studies and screening of additional patients for new mutations. Here we describe a novel *DNMT3A* germline variant identified by whole-exome sequencing in a patient with multiple paragangliomas and papillary thyroid carcinoma. The increased methylation of DNMT3A target genes observed in the proband’s sample points towards a gain-of-function effect of the variant, contrasting with the inactivation caused by loss-of-function alterations commonly seen in other neoplasia and in patients with Tatton-Brown-Rahman syndrome. This finding stresses the diverse molecular outcomes and suggests a heterogeneous phenotypic spectrum related to *DNMT3A* germline variants.

**Abstract:**

Over the past few years, next generation technologies have been applied to unravel the genetics of rare inherited diseases, facilitating the discovery of new susceptibility genes. We recently found germline *DNMT3A* gain-of-function variants in two patients with head and neck paragangliomas causing a characteristic hypermethylated DNA profile. Here, whole-exome sequencing identifies a novel germline *DNMT3A* variant (p.Gly332Arg) in a patient with bilateral carotid paragangliomas, papillary thyroid carcinoma and idiopathic intellectual disability. The variant, located in the Pro-Trp-Trp-Pro (PWWP) domain of the protein involved in chromatin targeting, affects a residue mutated in papillary thyroid tumors and located between the two residues found mutated in microcephalic dwarfism patients. Structural modelling of the variant in the DNMT3A PWWP domain predicts that the interaction with H3K36me3 will be altered. An increased methylation of *DNMT3A* target genes, compatible with a gain-of-function effect of the alteration, was observed in saliva DNA from the proband and in one independent acute myeloid leukemia sample carrying the same p.Gly332Arg variant. Although further studies are needed to support a causal role of *DNMT3A* variants in paraganglioma, the description of a new *DNMT3A* alteration in a patient with multiple clinical features suggests a heterogeneous phenotypic spectrum related to *DNMT3A* germline variants.

## 1. Introduction

The drip of new susceptibility genes involved in the development of pheochromocytoma (PCC) and paraganglioma (PGL), altogether PPGLs, has not ceased since the discovery of pathogenic variants in the succinate dehydrogenase (SDH) genes in the early 2000s. Thus, up to 35–40% of PPGL patients carry a germline variant in one of the almost twenty high—or low—penetrant susceptibility genes related to the disease described so far [1]. Although the clinical characteristics associated with alterations in the major susceptibility genes are well-known, most of the recently reported genes have been found mutated only in a few patients [2,3] and sometimes even in isolated cases [4]. In order to decipher the relevance of the new additions to this growing list of genes and to unravel specific genotype-phenotype associations, the description of additional cases is necessary.

One of the last members to be incorporated to the catalogue of genes associated with hereditary PPGL is the DNA methyltransferase 3 alpha (*DNMT3A*) gene [2]. Apart from a catalytically inactive regulatory orthologous, *DNMT3L*, mammals have two catalytically active DNA methyltransferases, DNMT3A and DNMT3B, in charge of the de novo generation of DNA methylation patterns during germ cell development and in the early embryonic phase [5,6]. Germline variants in *DNMT3A* are associated with developmental growth disorders, while somatic variants are frequently associated with malignancy. On the one hand, germline de novo loss-of-function variants in *DNMT3A* cause Tatton-Brown-Rahman syndrome (TBRS), an autosomal dominant condition characterized by overgrowth, intellectual disability and distinctive facial appearance [7]. On the other hand and despite not being identified until 2010 [8], somatic variants in *DNMT3A* are one of the most recurrent events across blood cancers of the myeloid lineage. Up to 22% of acute myeloid leukemia (AML) patients carry a somatic variant in *DNMT3A* [9,10] and these alterations have been associated with poor prognosis and adverse survival outcomes for AML patients. Interestingly, there is overlap of the spectrum of *DNMT3A* variants in TBRS and hematological malignancies [11], with some examples of both diseases occurring in the same patient [12,13]. Moreover, a germline *DNMT3A* loss-of-function variant has been found causing familial AML, in this case in the absence of TBRS symptoms [14].

We recently identified missense germline variants in *DNMT3A* in two patients with multiple head and neck PGLs [15]. Unlike what happens in patients with TBRS and in AML and other neoplasia, in which the gene is usually inactivated by loss-of-function alterations, we demonstrated that *DNMT3A*-mutated PGLs exhibited a significant overall methylation, indicating an activating role of the variants. A similar activation of *DNMT3A* was observed later on in patients with microcephalic dwarfism [16]. This duality has been also reported for the histone methyltransferase *EZH2*, in which somatic gain-of-function variants are frequently found in lymphoma [17,18], while inactivating germline alterations cause the Weaver overgrowth syndrome [19]. Herein, we describe and study an additional patient developing multiple head & neck PGLs and papillary thyroid carcinoma (PTC) who carries a new gain-of-function *DNMT3A* germline variant.

## 2. Results

### 2.1. Whole Exome Sequencing Analysis

The filtering process resulted in 541 variants, six of which were found in the public archive ClinVar (https://www.ncbi.nlm.nih.gov/clinvar/) (Table S1) and only one was found in a gene previously associated to PPGLs, *DNMT3A* (c.994G > A, p.Gly332Arg; VAF = 0.48). Interestingly, the only submission found in ClinVar regarding this variant was a de novo alteration identified in a patient with intellectual disability. The variant, subsequently validated by Sanger sequencing, was not found in 1942 population-matched unrelated Spanish exomes (http://csvs.babelomics.org/) and appeared in 3 of >140,000 exomes (allele frequency = 0.00001194) in the Genome Aggregation Consortium (gnomAD) database (http://gnomad.broadinstitute.org/). This frequency is below the frequency found for pathogenic variants in *SDHB*, a gene whose alterations have the lowest penetrance amongst those found in the known PPGL susceptibility genes. The *Gly332* residue is located within the Pro-Trp-Trp-Pro (PWWP) domain of DNMT3A, described to function as a chromatin methylation reader by recognizing both DNA and histone methylated lysines. There are 31 missense variants in gnomAD, including p.Gly332Arg, located within the PWWP domain of DNMT3A, all of them with frequencies bellow 6.4 × 10^−5^ (Table S2).

### 2.2. Presence of DNMT3A p.Gly332Arg in Databases and In Silico Predictions

**Figure 1:**
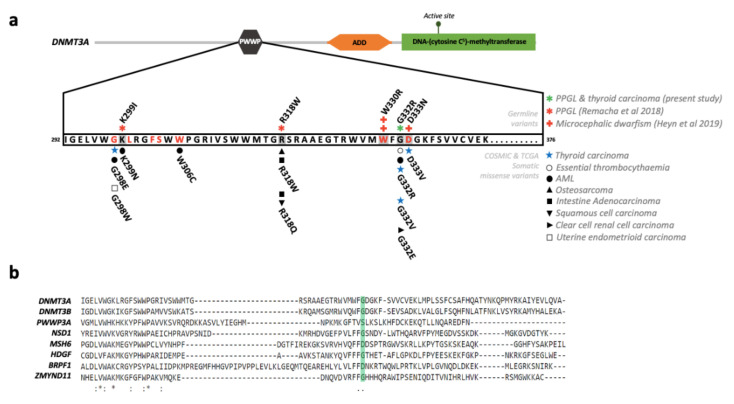
Missense variants and conservation of the PWWP domain of DNMT3A. (**a**) Representation of DNMT3A missense variants affecting the PWWP domain. Upper part, gain-of-function germline variants (denoted with a grey box) reported in patients with PPGL or microcephalic dwarfism. Bottom part, somatic variants reported in COSMIC (https://cancer.sanger.ac.uk/cosmic) and/or cBioPortal (www.cbioportal.org) databases affecting the same amino acids found mutated in the mentioned patients and/or affecting other residues involved in the aromatic cage [16,20] (denoted in red letters). (**b**) Multiple-sequence alignment of PWWP domains performed with Clustal Omega (European Bioinformatics Institute, Cambridge, UK). The alignment includes at least a member of each of the six classes of human PWWP domains since *DNMT3A* and *DNMT3B* share the same class (*DNMT3B* related) and *PWWP3A* and *HDGF* are also included in the same class (*HDGF* related) [20]. The position of DNMT3A-Gly332 is denoted with a green box.

The PredictSNP1 consensus classifier predicted the change to be deleterious (87% expected accuracy), Varsome (https://varsome.com) considered it as likely pathogenic and its frequency in gnomAD was lower than the threshold for *DNMT3A*. Three different missense somatic variants have been reported in five independent samples in COSMIC (https://cancer.sanger.ac.uk/cosmic) or cBioPortal (https://www.cbioportal.org/) databases affecting the *Gly332* residue (Figure 1a): p.Gly332Glu (*n* = 1; found in one clear cell renal cell carcinoma), p.Gly332Arg (*n* = 3; found in PTC, AML and essential thrombocythemia) and p.Gly332Val (*n* = 1; found in one PTC); although the causality of these variants has not been assessed.

**Figure 2:**
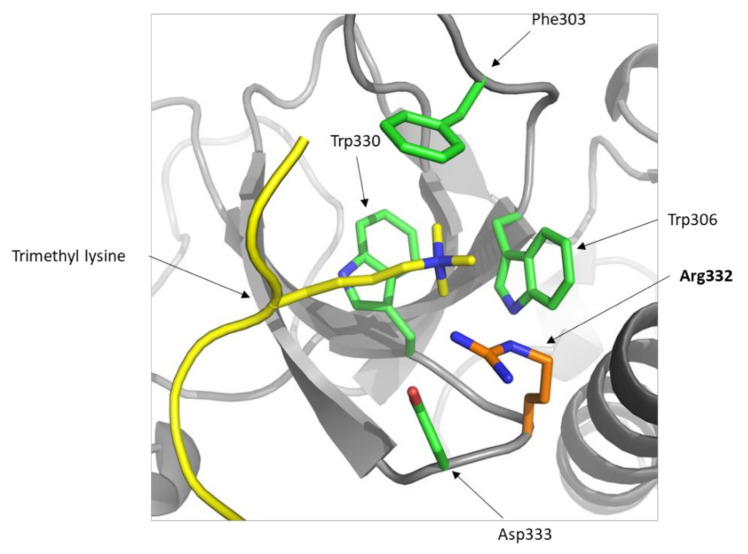
DNMT3A Gly332Arg mutant structure predicted with Phyre2. Trymethyl lysine (in yellow) is modelled from the DNMT3B structure (PDB code ID: 5CIU). The Arg332 mutant residue is colored in orange and the pivotal residues involved in the aromatic cage are colored in green and labeled.

Gly332 is conserved in four of the six classes of PWWP domains and the different residues found in this position have a weak similarity between them (Figure 1b). In addition, Gly332 is located in between Trp330 and Asp333, two pivotal residues for the aromatic cage of DNMT3A that recognizes tri-methylated lysine 36 of histone H3 (H3K36me3) (Figure 2). In the predicted structure of the PWWP domain of the DNMT3A Gly332Arg mutant, the position of the Arg lies opposite to the Phe303 residue and could form an additional side to the conserved aromatic cage formed by the amino acids Phe303, Trp306, Trp330 and Asp333. Thus, it is possible that an Arg residue in the 332 position could promote the stabilization of the binding to the trimethyl lysine present in histone H3.

### 2.3. Methylation Profiling

**Figure 3:**
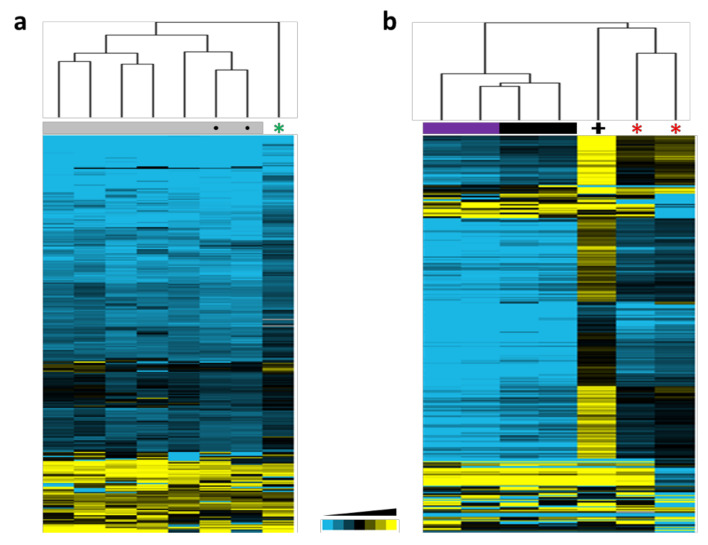
Hierarchical clusterings using methylation data from 307 probes found significantly differentially methylated between *DNMT3A*-mutated and non-mutated tissues in patients with PPGL [15]. (**a**) Unsupervised profiling using saliva DNA from the index patient (denoted with a green asterisk), in-home healthy controls (denoted with grey boxes and a dark circle) and healthy donors obtained from Gene Expression Omnibus (GEO) (denoted with grey boxes). (**b**) Profiling using methylation data from blood DNA obtained from two patients with PPGL (denoted with red asterisks) [15] compared to methylation data (GSE120428) [16] corresponding to: one patient with microcephalic dwarfism (denoted with a black cross), two patients with TBRS (denoted with purple boxes) and two controls (indicated with black boxes).

In order to obtain a representative methylation profile of *DNMT3A*-mutated tissues, we first got a list of 307 probes differentially methylated in *DNMT3A*-mutated tissues (Table S3). Then, we used this list to interrogate Illumina methylation data obtained from DNA saliva samples coming from the proband, two age/gender-matched controls and five healthy individuals. Hierarchical clustering grouped all the controls together and separated from the proband’s sample that showed a recognizable methylated profile (Figure 3a), suggesting that the p.Gly332Arg DNMT3A variant produces a similar alteration in germline global methylation than the observed for the previously reported PPGL patients.

We also speculated that the methylated profile observed upon alteration of *DNMT3A* in PPGL patients could be similar to the one described for patients with microcephalic dwarfism harboring germline gain-of-function *DNMT3A* variants. To explore this, we investigated the methylation status of the 307 differentially methylated probes in lymphocyte DNAs from the previously reported PGL *DNMT3A* variant carriers [15] and in blood samples from patients with either overgrowth or dwarfism due to *DNMT3A* germline variants [16]. Thus, we found that the blood DNA from PGL patients showed a similar profile and clustered together with the one obtained from the patient with microcephalic dwarfism (Figure 3b). The higher methylation observed in the patient with dwarfism suggests a higher impact of this variant in the activity of DNMT3A. Moreover, the mild differences observed between controls and the overgrowth patients suggest that the increased methylation caused by the gain-of-function probably affects non-canonical DNMT3A target regions unaltered in the presence of loss-of-function variants.

In addition, we were able to collect an AML sample carrying the p.Gly332Arg DNMT3A variant and investigated whether this somatic substitution could also be causing a gain-of-function in AML. To explore this, we profiled one AML sample carrying the p.Gly332Arg DNMT3A variant (this sample also carries another *DNMT3A* variant and two frameshift variants in *TET2*), with AML samples extracted from TCGA and carriers of *DNMT3A* somatic variants known to cause global methylation alterations (i.e., the bona fide p.Arg882 DNMT3A loss-of-function alteration). We also included three samples carrying additional frameshift *TET2* variants. The unsupervised clustering, using the aforementioned methylation signature, revealed that the p.Gly332Arg mutated AML showed a completely different profile to the one observed for the samples carrying the recurrent p.Arg882 DNMT3A inactivating variant (Figure S1). By including *TET2* mutated samples in the comparison, we discarded that this gene had an effect in the profiling. Moreover, we discarded a potential batch effect by performing a consensus clustering analysis with randomly selected sets of probes. Overall, these results suggest that the p.Gly332Arg DNMT3A variant led, in germline and also in AML, to a characteristic methylation profile, distinct to the profile caused by well-known *DNMT3A* inactivating variants. Although the sample carrying the p.Gly332Arg variant harbors an additional variant in the MTase domain of DNMT3A (p.Arg720Cys), its low allele frequency together with the fact that variants in this domain likely disrupt the catalytic activity of the enzyme, supports that the observed methylated profile is caused by the PWWP variant.

## 3. Discussion

Description of additional patients mutated in novel disease genes and comprehensive studies are required to advance in their clinical characterization. The link between global DNA hypermethylation and PPGL was first described in 2008, with the identification of a CpG island methylator phenotype (CIMP) in abdominal paragangliomas [21]. Later it was demonstrated that this was a consequence of oncometabolite accumulation, caused by pathogenic variants in Krebs cycle genes. More recently, disrupting variants in *ATRX* and in other chromatin-remodeling genes, including post-zygotic *H3F3A* gain-of-function alterations, have also been found recurrently in PPGLs [22,23]. The identification of *DNMT3A* activating variants leading to a specific CIMP further supported the association between epigenetic modifications and PPGL development.

In addition to the variants reported in patients with PGLs, germline gain-of-function *DNMT3A* variants located in the PWWP domain which cause widespread DNA hypermethylation at polycomb-regulated regions (with the H3K27me3 mark) have been found in patients with microcephalic dwarfism, an extreme growth disorder [16]. A similar growth deficiency has been observed in mice carrying a *DNMT3A* PWWP germline variant in the Asp329 residue (corresponding to the human Asp333). This variant causes a gain-of-function phenotype associated with the recruitment of DNMT3A towards H3K27me3-marked chromatin. This results in DNA hypermethylation and de-repression of developmental regulatory genes that manifests phenotypically as dominant postnatal growth retardation [24]. In the same way, the murine equivalent to the human Lys299Ile found in patients with PGL [15], disrupts both DNA and H3K36me2/3 binding by altering the aromatic cage conformation of the PWWP domain of DNMT3A, finally leading to disruption of the sub-nuclear localization of DNMT3A [25]. In PGL patients, we demonstrated that germline *DNMT3A* variants in residues within the PWWP domain caused significant hypermethylation of homeobox-containing genes involved in early embryonic development. However, long-term enforced expression of transgenic DNMT3A did not cause global hypermethylation of the genome [26]. This contrasts with the widespread hypomethylation observed in TBRS patients carrying germline *DNMT3A* variants, especially enriched at genes involved in morphogenesis, development and malignancy predisposition pathways [27].

Varsome classifies as likely pathogenic 74% (23/31) of the missense variants found in gnomAD within the PWWP domain of DNMT3A (Table S2). None of them appeared in TBRS patients and 58% (18/31) are found as somatic events in the COSMIC database, mostly affecting hematopoietic and lymphoid tissues. The conservation of Gly332 within the PWWP domain and its location between Trp330 and Asp333, the two residues altered in patients with microcephalic dwarfism [16], highly suggest a disruptive role of the variant. The substitution of the wild-type Gly332 by an Arg could stabilize the link with trimethylated histone H3 causing, as occurred with the previously reported variants found in PGL patients, a gain of alternative activities by DNMT3A.

Herein we have demonstrated that blood DNA from a patient with microcephalic dwarfism shows a similar profile to the observed for PGL patients, suggesting that a comparable activation of DNMT3A is occurring in both phenotypes. Moreover, overall alterations of DNA methylation have been also observed in blood cells from *TET2* (hypermethylation) or *EZH2* (hypomethylation) germline pathogenic loss-of-function variant carriers [28,29]. Thus, the methylated signature found in saliva DNA from the PGL patient described herein further supports the activating role of the new germline alteration found within the PWWP domain of DNMT3A. However and although the patient described herein showed idiopathic intellectual disability and short stature, there is no clear phenotypic overlap between PGL and microcephalic dwarfism. Interestingly, an AML pedigree carrying a germline *DNMT3A* loss-of-function mutation has no symptoms of TBRS and only three patients with TBRS have been reported to develop AML [14]. This could be suggestive of specific genotype-phenotype associations for some *DNMT3A* germline variants but there are other possible explanations such as the presence of additional modifier variants influencing the phenotypic outcome of these individuals.

Somatic alterations in the epigenetic regulators *TET2* and *DNMT3A* represent a frequent cause of clonal hematopoiesis, an aging-related mechanism in which somatic variants in early blood cell progenitors confer an advantage to mutated clones. Albeit asymptomatic, this mechanism is associated with a higher risk of cardiovascular disease and hematologic malignancies [30,31,32,33]. Interestingly, several variants affecting the Gly332 residue (including p.Gly332Arg and p.Gly332Glu) have been found in healthy individuals with clonal hematopoietic mutations [32,34,35]. In fact, the Gly332Arg variant is included in a list of leukemogenic driver mutations affecting genes known to promote clonal expansion of hematopoietic stem cells (Table S2). It has been suggested that the presence of some pathogenic TBRS variants amongst the general population is due to age-related clonal hematopoiesis, questioning the utility of databases such as gnomAD in *DNMT3A* variant pathogenicity stratification [13]. The methylated profile observed in the AML sample carrying the p.Gly332Arg variant further suggests that alteration of specific residues within the PWWP domain of DNMT3A leading to activation of its methylation capacities, may have oncogenic potential to both endocrine malignancies (PPGL) and AML.

Thyroid tumors show the second highest missense mutation rate (0.23; number of missense variants/number samples) affecting the DNMT3A PWWP domain amongst all cancers included in COSMIC, even higher than AML (0.13) (Table 1). Two recent studies described *DNMT3A* pathogenic variants occurring at a frequency of 1.2% (12/1013) in PTC [36] and associated with adverse clinical outcomes [37]. Interestingly thyroid toxic adenomas have been recently found in all mutation carriers belonging to a TET2 pedigree [38]. Both, adenomas and microcarcinomas, are precursor neoplasms of the thyroid suggesting a role of these epigenetic alterations in malignancy initiation [39]. Moreover, COSMIC reports only one *DNMT3A* variant affecting the microcephalic dwarfism-mutated residue Asp333 and it affects an anaplastic thyroid carcinoma [40], further supporting the relevance of the PWWP domain in thyroid cancer. All these findings suggest that *DNMT3A* alteration and the subsequent overall change in DNA methylation, plays an important role in thyroid pathogenesis as well, though the activating or inactivating nature of the alterations is still unknown. To note, AML, thyroid cancer and PPGL show the lowest tumor mutational burden amongst all neoplasia (*n* = 10–14) [41], something that indicates a low number of passenger variants and stresses the relevance for tumor development of mutated genes, such as *DNMT3A*.

In summary, we describe and perform methylome analysis for a novel *DNMT3A* activating variant in a patient with multiple clinical features. Although the causal role of *DNMT3A* variants in PPGL has not been demonstrated, our results stress the heterogeneous phenotypic spectrum related to *DNMT3A* germline variants and support a dual nature of this gene in disease.

## 4. Materials and Methods

### 4.1. Patients and Samples

A 38-year-old woman with idiopathic intellectual disability was remitted to the hospital with a cervical mass of three months of evolution. The initial diagnosis was multinodular goiter with cysts. Two years later, she underwent total thyroidectomy with a diagnosis of nodular hyperplasia and a papillary thyroid microcarcinoma of 0.5 mm and stage I (T1, N0, M0) that was subsequently treated with radioactive iodine (I-131). One year later, during the follow-up, two PGLs located in the right carotid (3 × 3.5 cm) and in the left vagus nerve were diagnosed by imaging and underwent surgical removal. Thirteen years later, a left carotid PGL (2.5 × 2.2 cm) was detected by computed tomography and echography. The tumor was not operated and, since then, the patient, who had no family history of thyroid cancer or PPGLs, has been subject to close follow-up of the established lesion. DNA from saliva was obtained from the patient and from two age- and gender-matched healthy controls, whereas tumor samples were not available. An informed consent was obtained from her legal guardian.

Tumor DNA from a patient with AML and carrying two somatic variants in *DNMT3A* (p.Gly332Arg and p.Arg720Cys) and two in *TET2* (p.Arg1216Ter and p.Arg544Ter) was used for methylation profiling.

### 4.2. Targeted Next-Generation Sequencing

Whole-exome sequencing was carried out at the National Centre for Genomic Analysis (CNAG) on DNA extracted from a saliva sample from the patient by OrageneTM self-collection kit (DNA GenoteK, Ontario, Canada). Exome capture was performed using the Roche NimbleGen exome capture kit (Roche NimbleGen, Madison, WI, USA) and exome sequencing (mean coverage > 80×) was performed by 2 × 100 base paired-end technology using a HiSeq2000 sequencer (Illumina Inc., San Diego, CA, USA). Alignment of the reads was performed by the GEM5 and BFAST programs and the SAMtools program was used (http://samtools.sourceforge.net) to identify single nucleotide substitutions and small insertions and deletions. Filtering was applied to exclude variants present in gnomAD with AF > 0.00005, variants present in internal exomes or in the CIBERER Spanish Variant Server (http://csvs.babelomics.org/), noncoding substitutions and variants in genomic regions with low mappability, with low depth readings or quality or with the alternative allele present in < 20% of reads.

### 4.3. In Silico Predictions

The PredictSNP1 consensus classifier [42] was used to predict the effect of the only nucleotide substitution affecting a gene previously associated with PPGL and that passed all filtering steps. The Clustal Omega program (https://www.ebi.ac.uk/Tools/msa/clustalo/) was used to generate an alignment of the six classes of human PWWP domains. The amino acid sequence of the DNMT3A PWWP domain (in which Gly332Arg mutant is located) was submitted to the Phyre2 server (http://www.sbg.bio.ic.ac.uk/phyre2) for structure prediction [43]. Twenty templates were selected to model the protein based on heuristics to maximize confidence, percentage identity and alignment coverage. The H3K36me3 peptide was modelled into the DNMT3A PWWP Gly332Arg mutant structure obtained using the DNMT3B PWWP domain structure bound to H3K36me3 peptide (PDB code 5CIU) [44] as a reference to ensure no clashes with the methylated lysine.

### 4.4. DNA Methylation Array

Bisulfite conversion of the AML tumor and the saliva DNAs from the proband and two age-and gender-matched controls was performed using the EZ DNA Methylation Kit (Zymo Research, Irvine, CA, USA) and genome-wide DNA methylation was assayed using the Infinium MethylationEPIC BeadChip (Illumina) at the Centro Nacional de Genotipado (CEGEN-ISCIII) (www.cegen.org), as previously described [45]. This BeadChip interrogates over 850,000 methylation sites per sample at single-nucleotide resolution. M values were used for statistical analyses.

For methylation analyses, first, supervised DNA analyses (using data from Reference [15]) were performed to obtain a list of differentially methylated probes (*n* = 307; false discovery rate (FDR) < 0.15) between *DNMT3A*-mutated tissues (blood and tumors) from patients with PGL and healthy controls (Figure S2). Following, we used this list of probes for profiling MethylationEPIC data obtained from the DNA saliva samples from the proband, the two age- gender-matched controls and five additional saliva samples of healthy donors obtained from Gene Expression Omnibus (GEO) (GSE111631). In a subsequent analysis, we used the same list of 307 probes to profile methylation data from blood of patients with microcephalic dwarfism (*n* = 1) or overgrowth syndrome (*n* = 2) (GSE120428) [16] and compare them to controls (*n* = 2) and to PGLs carrying *DNMT3A* variants (*n* = 2) [15]. Finally, we extracted methylation data from 12 AML samples available from The Cancer Genome Atlas (TCGA), corresponding to 213 out of the 307 probes aforementioned for hierarchical clustering with one additional AML sample carrying the p.Gly332Arg variant, another DNMT3A variant (p.Arg720Cys) and two frameshift *TET2* mutations. The TCGA samples were all carriers of mutations in DNMT3A known to cause global methylation alterations (i.e., p.Arg882Cys/His) and three of them also harbored *TET2* frameshift mutations. The reliability of the clusters was verified by consensus clustering, a robust clustering method which obtains the consensus across multiple runs of a clustering algorithm and assess the stability of the discovered clusters by using resampling techniques [46]. Hierarchical clustering of methylation data was performed using GeneCluster 2.0 [47].

## 5. Conclusions

We describe a novel germline *DNMT3A* variant (p.Gly332Arg) identified by whole-exome sequencing in a patient with multiple clinical features: bilateral carotid PGLs, PTC and idiopathic intellectual disability. The variant affects a residue found mutated in papillary thyroid tumors and the structural modelling of the PWWP domain predicts the substitution to alter the interaction of DNMT3A with H3K36me3. Moreover, we observed an increased methylation of DNMT3A target genes in saliva DNA from the proband and in one independent AML sample carrying the same p.Gly332Arg variant, compatible with a gain-of-function effect of the alteration. Although the causal role of *DNMT3A* variants in PPGL has not been demonstrated, our finding stresses the heterogeneous phenotypic spectrum related to *DNMT3A* germline variants.

## Figures and Tables

**Table 1:** Variants affecting the PWWP domain in cancers included in the COSMIC database.

Tissue	Number Samples Mutated	Samples Tested	% Samples with Mutations
Endometrium	7	958	0.730688935
Thyroid	5	2151	0.232450023
Large intestine	8	4480	0.178571429
Urinary tract	2	1204	0.166112957
Bone	1	724	0.138121547
AML	18	13,565	0.132694434
Skin	2	1780	0.112359551
Kidney	3	2790	0.107526882
Hematopoietic and lymphoid (≠ AML)	13	14,357	0.090548165
Lung	4	4931	0.081119448
Breast	4	5131	0.077957513
Biliary tract	1	1474	0.067842605
Central nervous system	2	3246	0.061614295
Esophagus	1	1668	0.059952038
Upper aerodigestive tract	1	1795	0.055710306
Liver	1	2370	0.042194093
Prostate	1	2869	0.03485535

## Supplementary Materials

The following are available online at https://www.mdpi.com/2072-6694/12/11/3304/s1, Figure S1: Hierarchical clustering performed with one AML sample carrying the p.Gly332Arg DNMT3A mutation (black circle) and 12 AML samples from TCGA [48] carrying mutations known to cause global methylation alterations: loss-of-function p.Arg882Cys/His DNMT3A mutations (blue boxes) and *TET2* frameshift mutations (purple boxes). Profiling was based on methylation data from the 213 probes, out of the 307 significantly differentially methylated between *DNMT3A*-mutated and non-mutated tissues in patients with PPGL, contained in the Infinium HumanMethylation450 BeadChip, Figure S2: Schematic representation of the methylation analysis pipeline, Table S1: Filtered exome variants found in ClinVar, Table S2: Variants affecting the PWWP domain found in gnomAD, Table S3: List of differentially methylated CpGs between *DNMT3A*-mutated and non-mutated samples.
