# Supplementary material for: Novel DNMT3A Germline Variant in a Patient with Multiple Paragangliomas and Papillary Thyroid Carcinoma
Source: Cancers (Basel). 2020 Nov 9;12(11):3304. doi: 10.3390/cancers12113304 (PMC7697455; doi:10.3390/cancers12113304)
Supplement: Supplementary file 1 [file cancers-12-03304-s001.pdf]

cancers

MDPI

Article

Novel *DNMT3A* Germline Variant in a Patient with Multiple Paragangliomas and Papillary Thyroid Carcinoma

Sara Mellid, Javier Coloma, Bruna Calsina, María Monteagudo, Juan M Roldán-Romero, María Santos, Luis J Leandro-García, Javier Lanillos, Ángel M Martínez-Montes, Cristina Rodríguez-Antona, Cristina Montero-Conde, Joaquín Martínez-López, Rosa Ayala, Xavier Matias-Guiu, Mercedes Robledo and Alberto Cascón

SupplementaryMaterials:

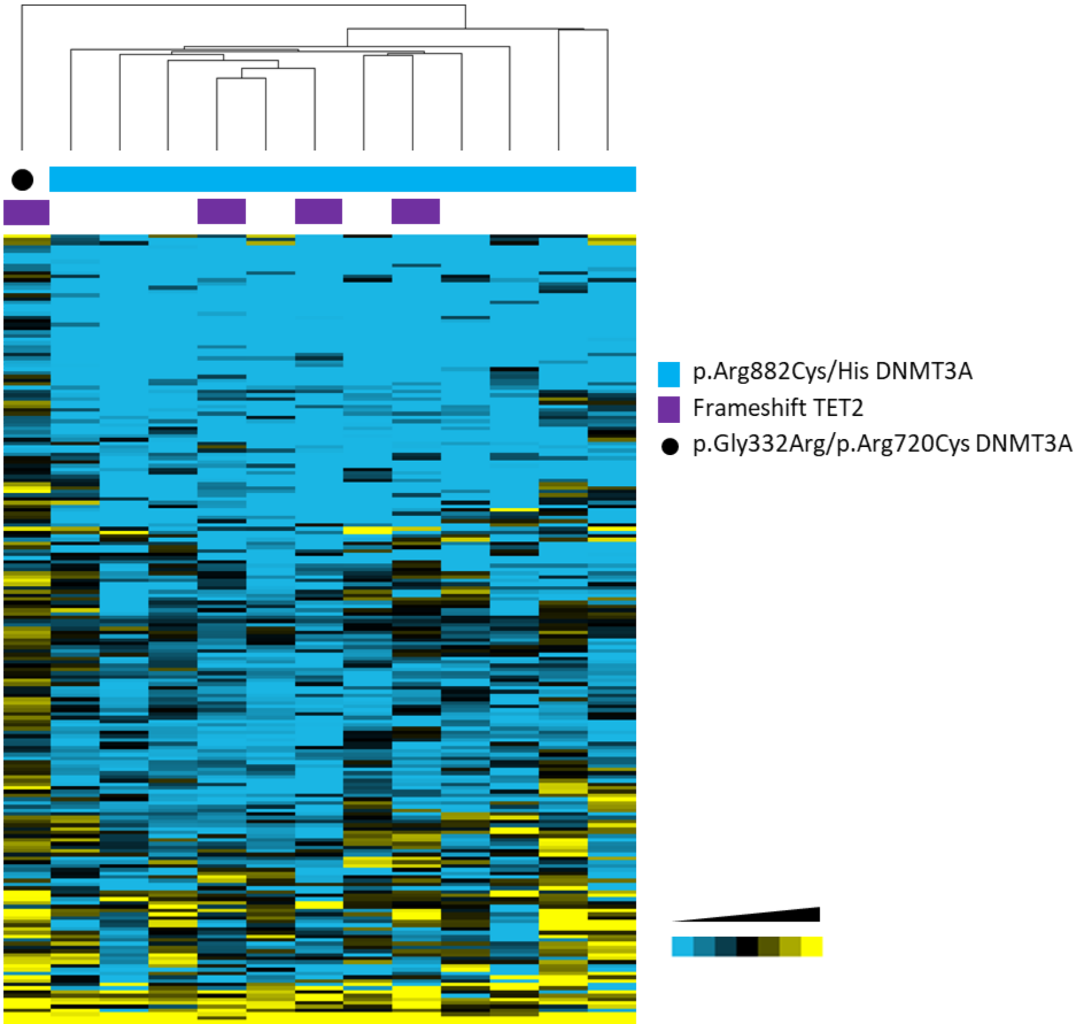

Figure S1. Hierarchical clustering performed with one AML sample carrying the p.Gly332Arg DNMT3A mutation (black circle) and 12 AML samples from TCGA [48] carrying mutations known to cause global methylation alterations: loss-of-function p.Arg882Cys/His DNMT3A mutations (blue boxes), and *TET2* frameshift mutations (purple boxes). Profiling was based on methylation data from the 213 probes, out of the 307 significantly differentially methylated between *DNMT3A*-mutated and non-mutated tissues in patients with PPGL, contained in the Infinium HumanMethylation450 BeadChip.

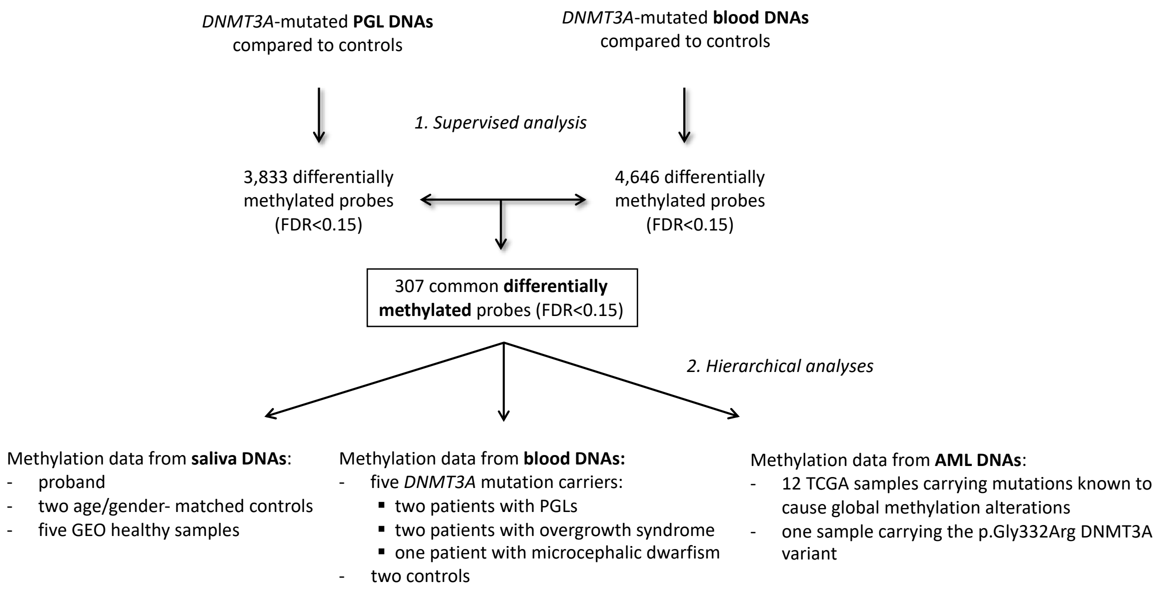

Figure S2. Schematic representation of the methylation analysis pipeline.

Table S1. Filtered exome variants found in ClinVar.

ClinVar ID	Chr	Coordinate	Gene Name	Transcript Conse-quence	Protein Consequence	dbSNP	PredictSNP1 (Confidence)	gnomAD Allele Frequency	Clinical Significance	Condition
VCV000519902	X	70339307	*MED12*	c.184G>A	p.Val62Ile	rs1039763693	Neutral (83%)	-	Uncertain	Cardiovascular phenotype
VCV000407388	4	55127414	*PDGFRA*	c.202G>A	p.Asp68Asn	rs1060501504	Neutral (83%)	0.0000239	Uncertain	Gastrointestinal stromal tumor
**VCV000981258**	**2**	**25470480**	***DNMT3A***	**c.994G>A**	**p.Gly332Arg**		**Deleterious (87%)**	**0.0000119**	**Uncertain**	**Intellectual disability**
VCV000284047	20	61467852	*COL9A3*	c.1571G>A	p.Arg524His	rs373382239	Neutral (74%)	0.0000252	Uncertain	Not provided
VCV000666692	12	15818756	*EPS8*	c.670G>A	p.Val224Ile	rs758506896	Neutral (83%)	0.0000887	Likely benign	Not specified
VCV000812325	3	50231196	*GNAT1*	c.460G>T	p.Asp154Tyr	rs1293620319	Neutral (72%)	0.00000413	Pathogenic	Retinitis pigmentosa

Table S2. Variants affecting the PWWP domain found in gnomAD.

	rsID	Transcript Consequence	Protein Consequence	Found in COSMIC	Found in AML in COSMIC	Leukemogenic Variant [1]	Varsome Prediction	Allele Frequency
1	rs777306476	c.875T>C	p.Ile292Thr	yes	yes	no	Likely Pathogenic	6,38162E-05
2	rs1402705749	c.887T>G	p.Val296Gly	yes	yes	no	Likely Pathogenic	3,98909E-06
3	rs1402705749	c.887T>A	p.Val296Glu	no	no	no	Likely Pathogenic	3,98909E-06
4	rs1410828051	c.893G>A	p.Gly298Glu	yes	yes	no	Likely Pathogenic	3,98746E-06
5	rs776841024	c.918G>T	p.Trp306Cys	yes	yes	no	Likely Pathogenic	3,98178E-06
6	rs759380437	c.920C>G	p.Pro307Arg	yes	yes	yes	Likely Pathogenic	3,98137E-06
7	rs759380437	c.920C>A	p.Pro307Gln	no	no	no	Likely Pathogenic	3,98137E-06
8	rs757575202	c.926G>C	p.Arg309Pro	yes	no	no	Likely Pathogenic	3,98105E-06
9	rs774128516	c.928A>T	p.Ile310Phe	no	no	no	Likely Pathogenic	3,97975E-06
10	rs747448117	c.976C>T	p.Arg326Cys	yes	yes	yes	Uncertain significance	3,97845E-05
11	rs747448117	c.976C>A	p.Arg326Ser	no	no	yes	Likely Pathogenic	7,95691E-06
12	rs758881009	c.977G>T	p.Arg326Leu	yes	no	yes	Likely Pathogenic	7,95684E-06
13	rs758881009	c.977G>A	p.Arg326His	yes	yes	yes	Likely Pathogenic	2,38705E-05
14	rs765593136	c.985A>G	p.Met329Val	yes	no	no	Uncertain significance	3,9789E-06
15	rs753896945	c.991T>G	p.Phe331Val	no	no	no	Likely Pathogenic	3,98004E-06
16	rs151221034	c.993C>G	p.Phe331Leu	no	no	no	Likely Pathogenic	3,9807E-06
17	rs760854242	c.994G>A	p.Gly332Arg	yes	yes	yes	Likely Pathogenic	1,19427E-05
18	rs1024296111	c.1000G>A	p.Gly334Ser	yes	no	no	Uncertain significance	3,98356E-06
19	rs773208295	c.1010C>T	p.Ser337Leu	no	no	no	Likely Pathogenic	1,19556E-05
20	rs749817324	c.1031T>C	p.Leu344Pro	yes	yes	yes	Likely Pathogenic	3,98819E-06
21	rs749817324	c.1031T>A	p.Leu344Gln	yes	yes	yes	Likely Pathogenic	3,18776E-05
22	rs1308281604	c.1037C>T	p.Pro346Leu	no	no	no	Likely Pathogenic	3,98559E-06
23	rs139053291	c.1055G>A	p.Ser352Asn	yes	yes	no	Likely benign	0,00004247
24	rs1326972729	c.1058C>T	p.Ala353Val	yes	no	no	Uncertain significance	0,000003982
25	rs1220753680	c.1082A>G	p.Lys361Arg	yes	no	no	Likely Pathogenic	0,000003981
26	rs774855118	c.1091T>C	p.Met364Thr	no	no	no	Uncertain significance	0,000003982
27	rs144062658	c.1094A>G	p.Tyr365Cys	no	no	no	Likely Pathogenic	0,000003983

28	rs767236033	c.1097G>A	p.Arg366His	no	no	yes	Likely Pathogenic	0,00001063
29	rs773941479	c.1106T>A	p.Ile369Asn	yes	no	no	Likely Pathogenic	0,000003985
30	rs371677904	c.1114G>A	p.Val372Ile	no	no	no	Likely benign	0,00002483
31	rs773722655	c.1115T>C	p.Val372Ala	no	no	no	Uncertain significance	0,000003991

[1] Bick et al 2020

Table S3. List of differentially methylated CpGs between DNMT3A-mutated and non-mutated samples.

TargetID	Chr.	Coordinate	GENE	logFC Tumors	FDR (DNMT3A-Tumors vs. Controls)	logFC Blood	FDR (DNMT3A-Blood vs. Controls)
cg10818676	1	167098094	DUSP27	6,358	0,031	4,377	0,056
cg17018201	6	33269769	TAPBP	5,908	0,077	4,274	0,056
cg16869349	18	35069156	CELF4	6,103	0,077	3,415	0,097
cg26444086	7	1162748	C7orf50	6,825	0,077	4,355	0,103
cg12088417	17	78574716	RPTOR	4,872	0,077	5,934	0,137
cg02099878	2	223176167		-5,756	0,077	-2,924	0,137
cg19080354	11	288021	ATHL1	-6,133	0,077	-2,782	0,137
cg05746069	5	153857907	HAND1	-4,361	0,077	-1,408	0,147
cg07397108	15	22562214		4,595	0,078	3,761	0,111
cg23121156	4	154710421	SFRP2	-4,353	0,078	-1,664	0,137
cg08886727	17	46801084	PRAC2	-4,767	0,078	-2,251	0,139
cg10824810	16	54967714	IRX5	-4,471	0,078	-1,594	0,142
cg05712748	1	43472312		6,026	0,078	4,417	0,056
cg02694427	2	176964512	HOXD12	-4,746	0,078	-1,755	0,137
cg19748684	2	175197431	SP9	-4,573	0,078	-1,823	0,141
cg01915516	1	3568243	TP73	-5,129	0,082	-1,491	0,137
cg06354054	12	125440391	DHX37	5,789	0,082	3,820	0,056
cg09684233	2	175206966		-4,483	0,082	-1,404	0,139
cg08216425	1	43476956		5,513	0,085	4,360	0,119
cg13125157	17	50237403	CA10	-4,878	0,087	-1,891	0,137
cg02668233	12	125813494	TMEM132B	4,885	0,091	3,265	0,069
cg10776061	19	12768390	MAN2B1	-4,965	0,091	-2,886	0,086
cg19151808	4	89619051	NAP1L5	-4,570	0,091	-4,169	0,097
cg08839358	2	38528761	ATL2	3,975	0,091	3,547	0,131
cg08307231	17	78642620	RPTOR	4,052	0,091	4,654	0,132
cg13085030	7	1090504	C7orf50	4,765	0,091	3,892	0,136
cg21907107	5	76932062	OTP	-4,075	0,091	-1,807	0,137
cg00072839	X	91035267	PCDH11X	-3,838	0,091	-2,212	0,137
cg05835105	4	111543401	PITX2	-4,543	0,091	-1,914	0,137
cg10375890	1	50892511		-3,839	0,091	-1,439	0,139
cg24914355	2	176959229	HOXD13	-4,795	0,091	-1,573	0,139
cg24181384	7	155302908	CNPY1	-3,815	0,091	-2,138	0,142
cg01326836	7	27224836	HOXA11-AS	-3,698	0,091	-1,408	0,142
cg02071600	5	78808852	HOMER1	-4,543	0,091	-1,606	0,144
cg02597299	5	151304559	GLRA1	-4,187	0,091	-1,810	0,147
cg17964510	2	175199694	SP9	-5,291	0,092	-2,732	0,126
cg20049415	20	21377671	NKX2-4	-3,855	0,092	-2,028	0,145
cg11904056	5	92918910	NR2F1	-3,534	0,092	-1,695	0,146
cg09289202	2	242443982	STK25	3,469	0,095	3,947	0,059
cg07570618	1	1992389	PRKCZ	4,259	0,095	4,252	0,086

TargetID	Chr.	Coordinate	GENE	logFC Tumors	FDR (DNMT3A-tumors vs. Controls)	logFC Blood	FDR (DNMT3A-Blood vs. Controls)
cg05394800	13	50707050	DLEU1	-4,241	0,095	-2,055	0,137
cg03086663	5	92918912	NR2F1	-3,991	0,095	-1,676	0,137
cg27210166	17	78574692	RPTOR	5,556	0,099	5,609	0,061
cg07592775	1	19210017	ALDH4A1	3,991	0,099	4,461	0,097
cg01061391	13	79168573	RNF219-AS1	-4,518	0,099	-2,985	0,097
cg18116968	7	25900668		-3,541	0,099	-2,356	0,126
cg03685272	6	100062830	PRDM13	-4,016	0,099	-2,225	0,131
cg22616881	7	96642096	DLX6-AS1	-3,520	0,099	-2,216	0,137
cg17358883	4	111559134	PITX2	-3,362	0,099	-1,816	0,137
cg22508145	19	17015427	CPAMD8	5,827	0,099	4,391	0,057
cg16471877	6	99817927	COQ3	4,271	0,099	4,642	0,071
cg08871399	12	62672755	USP15	3,953	0,099	4,387	0,111
cg07744432	19	20608532	ZNF826P	3,350	0,099	3,360	0,112
cg02878244	11	20181911	DBX1	-3,433	0,099	-1,884	0,137
cg10068417	13	50706583	DLEU1	-3,383	0,099	-1,757	0,137
cg25027832	2	176948544	EVX2	-4,416	0,099	-1,893	0,137
cg07201017	10	102996571	FLJ41350	-3,622	0,099	-1,752	0,137
cg01008088	12	131517623	ADGRD1	3,993	0,099	4,589	0,137
cg18442187	6	50794722	TFAP2B	-3,855	0,099	-1,663	0,137
cg11664139	2	175204249		-3,200	0,099	-1,842	0,137
cg08434574	6	99292438		-3,307	0,099	-1,886	0,137
cg00548708	17	48041547		-3,340	0,099	-2,343	0,137
cg02730303	11	103480630		-4,989	0,099	-1,780	0,137
cg02897537	10	124893832	HMX3	-3,274	0,099	-1,536	0,137
cg17495715	2	176950273		-4,057	0,099	-1,699	0,137
cg10776919	1	50889442	DMRTA2	-5,247	0,099	-1,601	0,137
cg18168844	5	50262917	CTD-2089N3.1	-3,356	0,099	-1,363	0,139
cg05196231	X	40944772	USP9X	-4,052	0,099	-3,293	0,140
cg23767994	7	96621323	DLX6-AS1	-3,282	0,099	-1,289	0,144
cg08238319	5	404713	AHRR	3,817	0,099	4,600	0,056
cg23348270	2	176969612	HOXD11	-3,801	0,099	-1,938	0,137
cg07875786	5	92918927	NR2F1	-3,519	0,099	-1,965	0,137
cg27583307	2	200320750	SATB2	-3,634	0,101	-1,307	0,142
cg17326555	1	119535693		-3,416	0,101	-1,630	0,143
cg01299579	2	10830716	NOL10	-3,468	0,101	-2,169	0,131
cg16044109	3	124762597	HEG1	3,358	0,101	1,713	0,137
cg00917569	16	51187388	SALL1	-3,320	0,101	-1,821	0,137
cg21726372	4	111540884	PITX2	-3,606	0,101	-2,165	0,137
cg11801727	1	228659093		-4,700	0,101	-2,484	0,111
cg04961582	14	95239464		-3,805	0,101	-2,183	0,119
cg08605326	17	50237401	CA10	-3,623	0,101	-1,720	0,137
cg11746813	12	54448090	HOXC4	-3,638	0,101	-2,013	0,137

TargetID	Chr.	Coordinate	GENE	logFC Tumors	FDR (DNMT3A-tumors vs. Controls)	logFC Blood	FDR (DNMT3A-Blood vs. Controls)
cg18109320	5	87985809	LINC00461	-3,340	0,101	-1,868	0,137
cg19883384	8	25905478	EBF2	-4,923	0,101	-1,858	0,137
cg27642784	3	157813670	SHOX2	-5,430	0,101	-2,621	0,137
cg04105760	2	175199722	SP9	-3,974	0,101	-1,700	0,139
cg23254393	4	85423132		-2,954	0,101	-1,383	0,143
cg06993307	16	54969051	CTD-3032H12.2	-4,310	0,101	-1,807	0,145
cg25397922	4	113431869		-4,513	0,102	-2,036	0,139
cg27002522	12	54426772	HOXC5	-3,301	0,103	-1,424	0,145
cg23506042	5	50262673	CTD-2089N3.1	-3,624	0,104	-1,559	0,147
cg15250633	17	80627638	RAB40B	3,624	0,104	3,844	0,083
cg22902288	7	925032	GET4	5,288	0,104	5,524	0,097
cg05056349	2	207629824	FASTKD2	-2,971	0,104	-2,231	0,131
cg18279094	1	63790044	FOXD3	-4,449	0,104	-2,128	0,131
cg08806496	5	92918943	NR2F1	-3,201	0,104	-2,133	0,131
cg05391318	1	200011684	NR5A2	-3,904	0,104	-1,549	0,137
cg06497668	2	45155703	RP11-89K21.1	-3,383	0,104	-1,558	0,137
cg18205770	5	87439353		-3,128	0,104	-1,531	0,137
cg01069159	5	161275865	GABRA1	-2,909	0,104	-1,491	0,138
cg01337207	6	32063835	TNXB	-3,085	0,104	-1,757	0,145
cg10441934	10	100995640	HPSE2	-3,638	0,104	-1,649	0,137
cg11719362	7	25900355		-3,591	0,104	-1,794	0,137
cg15074403	7	55141475	EGFR	3,749	0,106	3,656	0,056
cg13733314	7	20830816		-3,837	0,107	-1,656	0,137
cg00037457	17	59531744	TBX4	-3,074	0,108	-2,095	0,126
cg06199336	2	200329942	SATB2	-3,104	0,108	-1,677	0,137
cg04109990	9	12795679	LURAP1L	5,201	0,109	5,278	0,056
cg08187687	2	176943914	EVX2	-3,494	0,109	-1,427	0,137
cg09175792	10	50534598	C10orf71	4,496	0,110	4,489	0,101
cg10738003	11	120233535	ARHGEF12	-4,606	0,110	-2,402	0,126
cg13479204	17	46641708	HOXB3	3,701	0,110	2,770	0,137
cg06776712	5	54528369	CCNO	-2,894	0,110	-1,782	0,137
cg27625479	7	121943287	FEZF1	-3,216	0,110	-1,519	0,137
cg18443359	5	134374693	C5orf66	-2,827	0,110	-1,816	0,140
cg00529958	13	100624288	ZIC5	-2,881	0,110	-1,418	0,141
cg22072375	21	38081480	SIM2	-3,411	0,110	-1,993	0,144
cg27455578	7	86850357	TMEM243	-3,018	0,110	-1,461	0,139
cg24875029	2	105471438	POU3F3	-3,061	0,110	-2,030	0,129
cg00316839	4	85414135	NKX6-1	-5,019	0,110	-1,997	0,137
cg23634124	17	48049952	DLX4	-2,726	0,110	-1,251	0,146
cg27307729	5	50264628	CTD-2089N3.3	-2,833	0,111	-1,883	0,133

TargetID	Chr.	Coordinate	GENE	logFC Tumors	FDR (DNMT3A-tumors vs. Controls)	logFC Blood	FDR (DNMT3A-Blood vs. Controls)
cg01438365	17	59480470	TBX2	-2,738	0,111	-2,068	0,139
cg25593948	6	50786670	TFAP2B	-3,882	0,111	-1,639	0,139
cg04738965	3	147127662	ZIC1	-3,079	0,111	-1,415	0,147
cg03388193	10	100996070	HPSE2	-3,405	0,112	-1,545	0,138
cg21226224	8	55370171	SOX17	-3,781	0,112	-2,716	0,085
cg16247826	2	54614692		3,309	0,112	4,074	0,086
cg06523784	3	171870145	FNDC3B	3,611	0,112	2,730	0,137
cg21128569	2	176956396	HOXD13	-2,705	0,112	-1,737	0,137
cg18063495	5	50262796	CTD-2089N3.1	-3,076	0,112	-1,894	0,137
cg06925855	1	63792239	FOXD3	-3,536	0,113	-1,985	0,137
cg26153054	2	132152814		3,230	0,114	1,650	0,139
cg04636269	17	35300868	LHX1	-2,780	0,114	-2,026	0,131
cg14367020	6	99292286		-3,191	0,115	-2,570	0,103
cg01783394	5	50262941	CTD-2089N3.1	-2,878	0,115	-1,690	0,137
cg25226247	6	50785152	TFAP2B	-2,911	0,115	-1,630	0,137
cg17790928	7	1277790	UNCX	-3,075	0,115	-1,519	0,137
cg05090195	12	54379493	HOXC10	-3,833	0,115	-1,774	0,143
cg05836145	7	20827117	SP8	-2,776	0,115	-1,290	0,144
cg00705280	2	219866493	MIR375	-4,165	0,115	-2,356	0,144
cg07673740	4	85414194	NKX6-1	-3,089	0,115	-1,968	0,131
cg18451114	1	38511677	POU3F1	-3,683	0,115	-1,879	0,137
cg01825295	3	147131617	ZIC1	-2,956	0,115	-1,758	0,139
cg00586817	2	200329872	SATB2	-3,592	0,115	-1,811	0,137
cg19987665	2	223159250	PAX3	-2,577	0,116	-1,670	0,137
cg04613734	8	116663921	TRPS1	4,554	0,116	4,027	0,074
cg05578840	5	76934327	OTP	-4,606	0,116	-2,228	0,137
cg00316222	6	137817512		-2,793	0,116	-1,800	0,137
cg04388989	19	30715606		-2,701	0,117	-1,645	0,137
cg15353709	7	27238007	HOXA13	-3,891	0,117	-1,447	0,139
cg19264651	9	126780399	LHX2	-3,459	0,117	-1,742	0,139
cg01688609	19	1987279	BTBD2	3,460	0,118	5,176	0,111
cg00083018	6	101840329	GRIK2	-3,051	0,118	-1,356	0,137
cg11145899	3	128778725	GP9	3,629	0,118	2,431	0,144
cg03442378	11	44332385	ALX4	-3,336	0,118	-2,297	0,137
cg08138075	16	54968787	CTD-3032H12.2	-2,676	0,118	-1,757	0,137
cg21585766	13	100636870	ZIC2	-3,947	0,118	-1,544	0,137
cg18322569	1	91182777	BARHL2	-3,105	0,118	-2,011	0,137
cg04779860	10	131763929	EBF3	-2,664	0,118	-1,660	0,139
cg25609528	1	38510182	POU3F1	-3,166	0,118	-1,286	0,142
cg01524278	12	54355460		-2,728	0,118	-1,347	0,147

TargetID	Chr.	Coordinate	GENE	logFC Tumors	FDR (DNMT3A-tumors vs. Controls)	logFC Blood	FDR (DNMT3A-Blood vs. Controls)
cg18063312	5	72740737	RP11-79P5.6	-4,077	0,118	-2,586	0,112
cg01983682	2	162283979	SLC4A10	-2,791	0,118	-1,441	0,137
cg08693140	7	6653510	ZNF853	3,073	0,119	3,558	0,121
cg03555697	5	87983545	LINC00461	-2,624	0,119	-1,419	0,140
cg19944876	17	8905866		-2,654	0,119	-1,287	0,142
cg03119308	7	127950724	RBM28	2,765	0,119	3,280	0,068
cg17840250	6	32822857	PSMB9	4,173	0,119	4,491	0,121
cg06580725	13	100637930	ZIC2	-3,229	0,119	-2,089	0,126
cg05825073	7	27282237	EVX1	-3,284	0,119	-2,470	0,137
cg05380019	7	19146555	TWIST1	-3,250	0,119	-1,609	0,137
cg27565650	1	88151065		3,127	0,119	2,836	0,137
cg05875410	16	51187572	SALL1	-2,854	0,119	-1,567	0,140
cg09863450	7	19159534	AC003986.7	-2,928	0,119	-1,283	0,142
cg02600430	13	100623942	ZIC5	-2,736	0,119	-1,842	0,137
cg01376826	3	147129826	ZIC1	-3,852	0,120	-2,017	0,137
cg01708273	2	176974060	HOXD11	-2,614	0,121	-1,234	0,144
cg06440305	20	21378427	NKX2-4	-2,835	0,122	-2,050	0,126
cg08545493	6	6004762	NRN1	-2,770	0,122	-1,699	0,149
cg01557547	3	157823089	SHOX2	-3,057	0,122	-1,557	0,142
cg26400885	3	147126119	ZIC4	-3,019	0,122	-1,251	0,143
cg24104938	6	108487078	NR2E1	-2,815	0,122	-1,464	0,137
cg26296371	6	5442953	FARS2	3,461	0,123	4,328	0,131
cg21849844	13	100611865		-3,121	0,123	-1,677	0,137
cg10393811	2	176983927	HOXD10	-3,427	0,123	-2,116	0,137
cg00040007	15	41222276	DLL4	-2,963	0,123	-1,374	0,147
cg23946695	6	1609223	FOXC1	-2,773	0,123	-2,239	0,137
cg19515484	6	1606877	FOXC1	-3,410	0,123	-1,369	0,139
cg06134910	10	26730561	APBB1IP	3,380	0,125	4,130	0,056
cg01414116	12	130720805		4,550	0,125	3,773	0,062
cg01961105	16	54968783	CTD-3032H12.2	-3,594	0,125	-1,611	0,137
cg18322025	6	170055332	WDR27	-2,807	0,125	-2,436	0,137
cg10032131	11	57283055	SLC43A1	-3,545	0,125	-5,466	0,085
cg13691003	1	75600399	LHX8	-3,268	0,125	-1,372	0,138
cg26678970	13	79171469	RNF219-AS1	-2,675	0,125	-2,551	0,111
cg26489108	9	975856	DMRT3	-3,021	0,125	-1,761	0,137
cg03958979	6	108486387	NR2E1	-2,685	0,126	-1,644	0,137
cg19025113	2	45155201	RP11-89K21.1	-2,625	0,126	-1,644	0,137
cg15425541	14	61118751	SIX1	-3,667	0,126	-2,580	0,137
cg09492451	16	54967389	IRX5	-3,153	0,126	-1,234	0,145
cg09739413	6	32729498	HLA-DQB2	-2,696	0,126	-1,351	0,148

TargetID	Chr.	Coordinate	GENE	logFC Tumors	FDR (DNMT3A-tumors vs. Controls)	logFC Blood	FDR (DNMT3A-Blood vs. Controls)
cg12399700	12	113902262	LHX5	-3,095	0,126	-1,898	0,137
cg08767182	5	92940163		-3,050	0,126	-1,712	0,143
cg02245004	15	76634887	RP11-685G9.2	-2,736	0,126	-1,316	0,147
cg09919570	16	49311782	CBLN1	-2,545	0,126	-1,614	0,138
cg20785796	2	200326721	SATB2	-2,505	0,127	-1,682	0,137
cg08600862	5	76939558		-2,961	0,127	-1,512	0,137
cg21592065	1	50889124	DMRTA2	-2,689	0,127	-1,490	0,137
cg02067712	13	28364724		-3,128	0,127	-1,520	0,138
cg05387399	10	124893766	HMX3	-3,256	0,128	-2,076	0,126
cg18277979	9	79630127		-2,482	0,128	-1,596	0,137
cg18606375	5	134365728	PITX1	-2,939	0,128	-1,356	0,139
cg14106680	19	50733985	MYH14	-3,082	0,128	-2,743	0,137
cg18468917	12	47353065		2,668	0,130	4,015	0,056
cg05640538	9	126772210	LHX2	-2,920	0,130	-1,586	0,137
cg23120061	2	176951595		-2,579	0,130	-2,679	0,145
cg17939889	2	240174650	HDAC4	2,541	0,130	1,347	0,143
cg08364561	11	31846844	RCN1	-2,844	0,130	-2,827	0,137
cg23934731	15	89904873	CTD-2335A18.1	-3,899	0,130	-1,560	0,137
cg11604182	5	177433673	FAM153C	-2,521	0,130	-1,376	0,138
cg13692446	13	112759719		-3,725	0,131	-1,733	0,137
cg17222645	3	197808017	ANKRD18DP	-2,662	0,131	-2,479	0,137
cg23698978	7	20838631		-3,278	0,131	-1,619	0,137
cg27069921	5	54523727	RP11-528L24.3	-3,121	0,132	-1,580	0,137
cg02973171	11	82443614	FAM181B	-3,606	0,132	-1,924	0,138
cg08880082	14	90165664	RP11-33N16.3	2,665	0,133	4,192	0,056
cg27299712	1	6550532	PLEKHG5	2,920	0,133	2,830	0,142
cg07747970	17	35293719	LHX1	-2,555	0,133	-1,264	0,147
cg14527649	14	69412803	ACTN1	3,269	0,134	3,570	0,137
cg03465861	15	76630660	ISL2	-3,301	0,134	-1,502	0,147
cg14775296	2	66672841	MEIS1	3,126	0,134	1,832	0,150
cg07335343	5	78829309	Y_RNA	3,464	0,135	4,541	0,120
cg13496838	2	223164747	PAX3	-3,019	0,135	-1,637	0,137
cg04591032	17	46827458		-2,472	0,135	-1,552	0,140
cg20268039	7	25900167		-2,843	0,135	-1,376	0,147
cg04655510	20	21378703	NKX2-4	-3,126	0,135	-1,644	0,137
cg24616539	11	31848828	RCN1	-2,734	0,135	-1,140	0,149
cg26006870	8	11559039	GATA4	-2,453	0,135	-1,271	0,142
cg11342452	10	134600463	NKX6-2	-3,100	0,135	-1,940	0,137
cg07476673	6	170602968	FAM120B	-2,403	0,135	-1,245	0,141

TargetID	Chr.	Coordinate	GENE	logFC Tumors	FDR (DNMT3A-tumors vs. Controls)	logFC Blood	FDR (DNMT3A-Blood vs. Controls)
cg00565688	1	3568212	TP73	-4,892	0,136	-1,452	0,137
cg08787268	17	47072857	RP11-501C14.5	-2,888	0,136	-1,522	0,142
cg27234067	11	32460799	WIT1-AS	-2,356	0,136	-1,871	0,142
cg11382529	8	65490613	LOC401463	-3,357	0,136	-2,314	0,137
cg21781761	19	20608600	ZNF826P	2,324	0,137	3,174	0,106
cg02288088	2	119615605		-3,212	0,137	-2,178	0,131
cg08463788	1	197888068	LHX9	-2,645	0,137	-1,660	0,137
cg20012315	7	6740598	ZNF12	3,583	0,137	3,590	0,071
cg03449867	15	28200653	OCA2	-4,501	0,137	-2,052	0,137
cg10425754	16	49311483	CBLN1	-2,516	0,137	-1,543	0,138
cg08460041	1	165325136	LMX1A	-2,554	0,137	-1,469	0,142
cg09890891	15	96904723	RP11-522B15.3	-2,454	0,137	-1,378	0,145
cg07699771	8	1153329	CTD-2281E23.2	-2,487	0,137	-1,495	0,137
cg00014830	17	77765733		-3,437	0,137	-1,508	0,137
cg26327666	5	76937963		-2,431	0,137	-2,134	0,139
cg04087742	5	50264462	CTD-2089N3.3	-2,508	0,138	-2,514	0,140
cg22239201	4	95678703	BMPR1B	-3,147	0,138	-1,223	0,142
cg17450425	8	70982255	PRDM14	-2,454	0,138	-2,437	0,137
cg11855516	2	175207244		-3,753	0,138	-1,662	0,137
cg04130163	4	42401214	SHISA3	-2,430	0,138	-1,239	0,142
cg25221239	10	131764419	EBF3	-3,148	0,138	-1,167	0,148
cg11988733	2	176968814	HOXD11	-2,392	0,138	-1,814	0,137
cg05022105	11	32460980	WT1	-2,830	0,138	-2,212	0,117
cg01582980	7	20825545	SP8	-2,386	0,138	-1,290	0,144
cg15254559	7	27292145		-2,369	0,139	-1,183	0,148
cg15301794	11	31822243	PAX6	-2,418	0,139	-2,052	0,137
cg07723251	17	50237742	CA10	-4,331	0,139	-1,482	0,137
cg09251429	11	124735128	ROBO3	-3,405	0,139	-1,572	0,140
cg17733447	17	45929399	SP6	-2,624	0,139	-1,608	0,137
cg18383890	13	100636252	ZIC2	-2,659	0,140	-2,181	0,126
cg09793883	1	91300559	RP4-665J23.1	-2,900	0,140	-1,834	0,137
cg02153806	7	96641232	DLX6-AS1	-3,112	0,140	-1,683	0,137
cg00645884	10	6110377		2,574	0,140	1,924	0,139
cg07339662	7	1271925	UNCX	-2,305	0,141	-1,292	0,148
cg10666140	7	855006	SUN1	-3,207	0,141	-1,295	0,148
cg24051749	1	39340282	MYCBP	3,408	0,141	4,361	0,056
cg14964115	10	116634877	FAM160B1	-3,499	0,141	-3,311	0,111
cg17875555	8	23564490	NKX2-6	-3,171	0,142	-2,070	0,131
cg26824678	1	19777949	CAPZB	3,739	0,142	4,016	0,071

TargetID	Chr.	Coordinate	GENE	logFC Tumors	FDR (DNMT3A-tumors vs. Controls)	logFC Blood	FDR (DNMT3A-Blood vs. Controls)
cg13023870	14	37124018	RP11-964E11.2	-3,666	0,143	-1,582	0,137
cg05425699	7	47580148	TNS3	3,325	0,143	1,382	0,148
cg23468312	8	130426887	CCDC26	2,232	0,144	1,760	0,137
cg05301866	2	162279964	TBR1	-2,278	0,145	-1,479	0,137
cg17833476	5	170736201	TLX3	-3,525	0,145	-1,885	0,140
cg00931201	X	15756372	CA5B	-2,326	0,146	-2,284	0,131
cg09162333	2	19556876	OSR1	-3,164	0,146	-2,004	0,137
cg24697433	6	28876508	TRIM27	3,289	0,146	4,648	0,056
cg01998213	7	19149989	TWIST1	-3,169	0,146	-1,592	0,137
cg15920743	10	102984393		-2,784	0,146	-1,438	0,137
cg27268520	6	27198236		-2,291	0,146	-2,505	0,137
cg13343639	10	100995599	HPSE2	-2,311	0,146	-1,632	0,137
cg03261929	13	79168211	RNF219-AS1	-2,763	0,147	-1,727	0,137
cg26365545	5	76932016	OTP	-2,406	0,147	-1,829	0,137
cg02573472	5	76940356		-2,629	0,147	-1,612	0,137
cg07474083	16	3225553		-2,259	0,147	-1,858	0,143
cg06058170	5	78808668	HOMER1	-3,205	0,148	-1,512	0,145
cg04184019	4	85424288		-2,555	0,148	-1,731	0,137
cg16810310	11	66104993	BRMS1	2,703	0,148	3,552	0,056
cg24851478	3	62355150	RP11-204J18.3	-2,810	0,148	-1,182	0,144
cg12421755	15	53080933	ONECUT1	-2,582	0,148	-1,813	0,137
cg03665912	X	136511413		-2,240	0,149	-1,696	0,137
cg18161327	13	100627703	ZIC5	-3,080	0,149	-1,725	0,142
cg14388488	20	61051777	GATA5	-2,249	0,149	-1,709	0,145
cg11841038	6	1380898		-2,231	0,149	-1,622	0,137
cg17486263	1	200011726	NR5A2	-2,168	0,150	-1,852	0,142
cg02194243	16	54324597	IRX3	-3,812	0,150	-1,960	0,137

© 2020 by the authors. Licensee MDPI, Basel, Switzerland. This article is an open access article distributed under the terms and conditions of the Creative Commons Attribution (CC BY) license (http://creativecommons.org/licenses/by/4.0/).
